# Photobiomodulation Therapy in the Treatment of Oral Mucositis, Dysphagia, Oral Dryness, Taste Alteration, and Burning Mouth Sensation Due to Cancer Therapy: A Case Series

**DOI:** 10.3390/ijerph16224505

**Published:** 2019-11-15

**Authors:** Marwan El Mobadder, Fadi Farhat, Wassim El Mobadder, Samir Nammour

**Affiliations:** 1 Department of Dental Science, Faculty of medicine, University of Liège, 4000 Liège, Belgium; S.Namour@ulg.ac.be; 2 Department of Hematology-Oncology, Hammoud Hospital University Medical Centre, Saida 652, Lebanon; drfadi.research@gmail.com (F.F.); wmobader@gmail.com (W.E.M.)

**Keywords:** cancer complications, dysphagia, dysgeusia, oral dryness, supportive cancer care, taste alteration

## Abstract

Oral complications of cancer therapy, such as oral dryness, dysphagia, and taste alteration, are associated with a negative impact in the quality of life of the patients. Few supportive care measures are available for such complications. This case series reveals the effectiveness of the photobiomodulation (PBM) therapy when used in a specific protocol and parameters, in the management of oral complications related to cancer therapy. Dysphagia was measured using the functional outcome swallowing scale for staging oropharyngeal dysphagia (FOSS). Oral mucositis was measured according to the National Cancer Institute scale. The quantity of the whole resting and stimulated saliva was measured in order to assess the oral dryness. In addition, the taste alteration was measured according to a protocol suggested by the International Standards organization (ISO). Sensation of burning mouth was measured using a visual analogue scale. These measurements were made before treatment, during, and at the end of the treatment. Diode laser 635 nm was used in 3 J/cm^2^. Five sessions interleaved with 24 h breaks were conducted for the dysphagia and oral dryness, and 10 sessions were conducted for the taste alteration and burning mouth sensation. Regardless of the limitations of this case series, PBM can be considered safe, time saving, and a promising approach for the management of the oral complications due to cancer therapy and the quality of life of cancer patients.

## 1. Introduction

Photobiomodulation (PBM) therapy (photon and biological modulation) is a therapeutic approach that modulates biological activity by employing light at red and near-infrared wavelengths [1,2,3]. The North American Association of Laser Therapy (NAALT) and the World Association of Laser Therapy (WALT) reached a consensus in 2014 on the nomenclature of photobiomodulation (PBM) as the therapeutic use of light [4,5]. The first evidence of the biostimulation effect of the lasers dates back to 1967 in an experiment by Andre Mester [6]. In recent years, the application of PBM has moved on rapidly due to the combination of a better understanding of the technical, clinical, and photobiological principles of the use of red and infrared light [7]. At present, a great number of studies suggest that PBM significantly reduces inflammation process, reduces pain, prevents fibrosis, and enhances wound healing and tissue regeneration [7,8]. Although there is a surfeit of studies evidencing that PBM effectively modifies biological functions, the complex biologic mechanism PBM exerts its therapeutic effects with has not been fully understood, where it varies according to different tissue states, cell type, irradiation parameters, and other factors [9]. PBM was shown to act primarily by increasing the ATP production and causing a short burst of reactive oxygen species (ROS) [10]. The most acceptable theory is that cytochrome c oxidase (CcO) by the red and infrared light will cause an increase in the ATP production [10,11,12]. In addition, recent studies have suggested that PBM may activate transcription factors and signaling pathways and may have a protective mechanism [10,11,12].

It is confirmed in literature that chemotherapy (CT) and/or head and neck radiation therapy (HNRT) can have tremendous negative impact on the quality of life of the patients and can largely affect their adherence to the treatment [13,14]. One of the most common oral complication known due to cancer therapy is the inflammation of the oral mucosa defined as oral mucositis (OM) [15,16]. The national cancer institute (NCI) defines oral mucositis as an acute inflammation and/or ulceration of the oral or oropharyngeal mucosal membranes. It can cause pain/discomfort; interfere with eating, swallowing, and speech; and may lead to infection. The severity of OM can vary from discomfort from erythema and soreness to severe ulcers that make alimentation impossible [15,16]. In addition to the oral mucositis, patients experience significant alteration in swallowing (dysphagia), alterations in taste perception (dysgeusia), hyposalivation, oral dryness, osteonecrosis of the jaw, trismus, speech alteration, as well as chronic pain [17,18,19]. According to the World Gastroenterology Organization (WGO), dysphagia refers either to the difficulty someone may have with the initial phases of a swallow or to the sensation that foods and or liquids are somehow being obstructed in their passage from the mouth to the stomach.

These oral complications are associated with a high possibility of a negative impact not only on the quality of life (QoL) but also on the patient’s compliance to therapy and the clinical outcomes; this is why it is important to prevent and to treat these complications [19]. On the other hand, there are only few available measures to prevent and/or treat these oral complications of cancer therapy, and to the best of our knowledge, very few investigations studied the use of PBM in the treatment of dysphagia, oral dryness, and taste alteration due to cancer therapy [20].

The aim of this case series is to assess the effectiveness of photobiomodulation therapy with a specific protocol that was suggested by a multinational panel of experts in the field of photobiomodulation and supportive care in cancer patients in the treatment of dysphagia, oral dryness, taste alteration, and burning mouth sensation [19].

## 2. Case Reports

### 2.1. Case 1: Oral Dysphagia and Oral Mucositis

**Figure 1:**
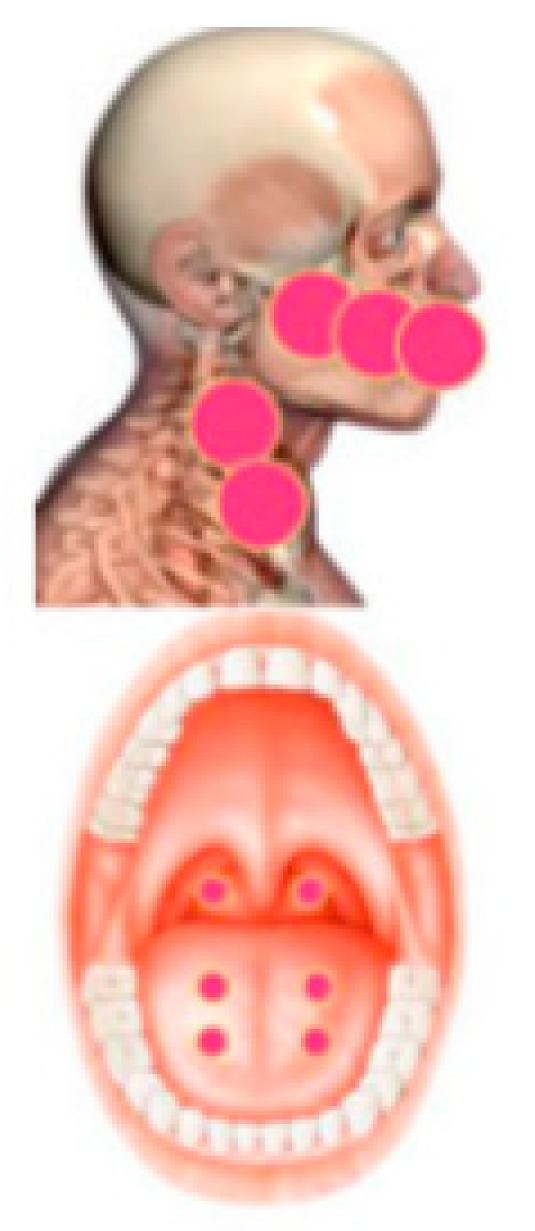
Photobiomodulation (PBM) treatment area for the management of oral mucositis [19].

A 59-year-old woman with breast cancer, under Everolimus medication, was brought to the clinic complaining of a chronic swallowing impairment (dysphagia) and pain sensation with a feeling of generalized hot oral mucosa. The patient signed a written informed consent before her engagement in the study. The patient was under 5 mg/day Everolimus (Afinitor) for 2 months. According to the patient, the symptoms appeared the first month of medication intake and persisted. The clinical examination revealed the presence of erythema and ulcers, but these did not interfere with the patient’s diet. According to a meticulous examination of the oral cavity, the patient was diagnosed with oral mucositis grade II of the national cancer institute scale (NCI) (Table 1). A speech therapy specialist diagnosed the patient with a chronic dysphagia. The NCI assessment scale for oral mucositis and the functional outcome swallowing scale for staging oropharyngeal dysphagia (FOSS) (Table 2) [21] were used in order to assess the severity of the complication before the treatment and after 24 h of each treatment. According to the FOSS scale, the patient showed a compensated abnormal function manifested by significant dietary modifications and prolonged mealtime with a stable weight and occasional cough with an absent aspiration—therefore a stage II of the FOSS scale [21]. According to the oral mucositis assessment scale for OM, the patient was diagnosed with a stage 2. The treatment of choice was the therapeutic use of photobiomodulation therapy. For the management of oral mucositis, diode laser 635 nm (smart M Pro, Lasotoronix, Poland) was intraorally applied at energy density of 3 J/point and a time of 30 s, output power of 100 mW, in a continuous and contact mode on four points on the tongue and two on the oropharynx (Figure 1). Extraoral application of diode laser was conducted with a wavelength of 635 nm, energy density of 3 J/point, output power of 100 mW, and a time of 30 s per point on the following areas: Lips, cutaneous surface corresponding to the buccal mucosae, and bilateral cervical lymphatic chain (Table 3). For the management of dysphagia, the parameters were as follows: Diode laser 635 nm (smart M Pro, Lasotronix, Poland) 3 J/cm^2^ for 30 s on each point, output power of 100 mW, continuous and contact mode. One session of PBM was conducted each 24 h for five days. The intraoral irradiated surfaces were bilaterally: Four points on the soft palate and four points on the oropharynx. The extraoral irradiated surfaces were lateral and ventral pharynx and larynx, midline neck, and lateral neck anterior to sternocleidomastoid muscle (Table 4). After treatment, a significant reduction of dysphagia (from stage II to Stage 0) was noted and a significant reduction of the oral mucositis was noted (Table 5). Therefore, PBM therapy successfully treated the cancer therapy-induced dysphagia.

### 2.2. Case 2: Oral Dryness

**Figure 2:**
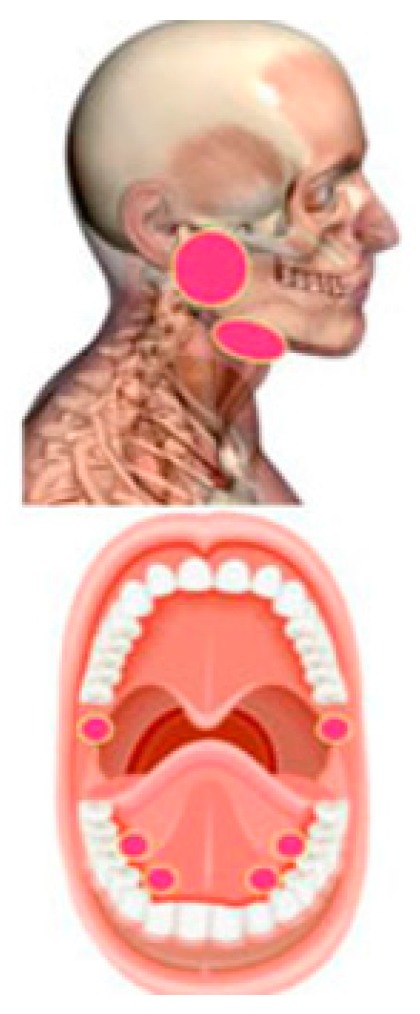
PBM treatment area for the management of oral dryness [19]

A 48-year-old male patient diagnosed with adenocarcinoma consistent with salivary duct carcinoma underwent intensity-modulated radiation therapy for two months and was referred to the clinic. The patient signed a written informed consent before his engagement in the study. During the high dose radiation therapy, the patient started to complain of a chronic oral dryness that persisted over time. According to the patient, the oral dryness persisted with no improvement with time. Based on a meticulous clinical examination, oral dryness due to irritation of the major salivary glands was diagnosed. The treatment of choice was photobiomodulation therapy each 24 h for five sessions. Diode laser 635 nm (smart M Pro, Lasotronix, Poland) was used with the following parameters: Energy of 3 J/cm^2^, output power of 100 mW, time of irradiation of 30 s on each point (Figure 2), continuous and contact mode (Table 6). In order to assess the severity of oral dryness and to measure the impact of the treatment, quantity of resting and stimulated saliva before and after stimulation was measured. Expectoration of all saliva into a graduated test tube was conducted for a 10-minute period without stimulation. After citric acid stimulation, the patient was also invited to expectorate all the saliva for only 5 min. After 24 h of each session, the measurements of the resting and stimulated saliva were made. This method used to assess the severity and the variation of oral dryness has been suggested by a systematic review [22]. The quantity of the resting and stimulated saliva increased significantly after the treatment (Table 7). According to these findings, PBM effectively increases the salivation.

### 2.3. Case 3: Taste Alteration Associated with Burning Mouth Sensation

**Figure 3:**
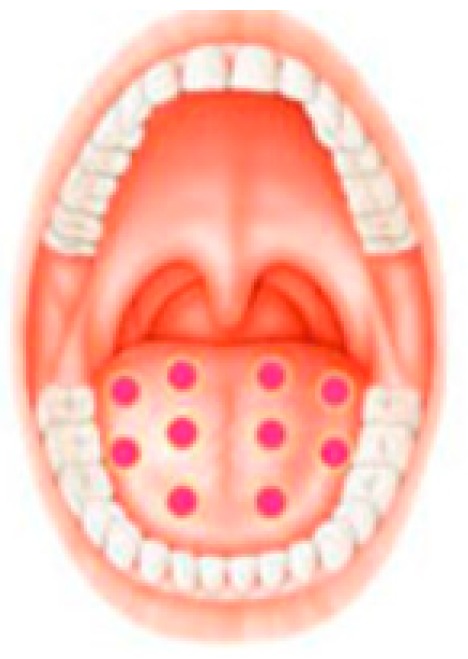
PBM treatment area for the management of taste alteration [19].

A 42-year-old man underwent intensified head and neck radiotherapy and was referred to the clinic with a chief complaint of a complete loss of taste function and a sensation of mouth burning. The patient signed a written informed consent before his engagement in the study. According to a thorough clinical examination, the diagnosis was a taste alteration due to direct neurological toxicity of the taste buds cells of the tongue. In order to assess the severity of the taste alteration, the International Standards Organization (ISO) ISO 3972:2011 for the measurements of taste alteration was used. Sweet, salty, sour, bitter, and umami were each tasted in a single “sip and spit” technique after a rinse of the mouth with room-temperature, purified water three times before and after sampling and expectorating each solution. The solutions and their corresponding concentrations were sucrose 300 mM, NaCl 200 mM, citric acid 5 mM, caffeine 10 mM, and monosodium glutamate (MSG) 200 mM. Perceived taste quality was identified by selecting one of seven choices. Correct responses were sweet for sucrose, salty for NaCl, sour for citric acid, bitter for caffeine and savory for MSG. Further choices were none or metallic. The score was assigned as 0–5 correct choices—if the patient failed to identify the correct taste (0) and if the answer was correct (1). Before any examination and data collecting, the patient was asked to stop eating and to drink only water at least one hour prior to testing. The taste alteration score was zero out of five before treatment. In addition, in order to assess mouth-burning sensation, visual analogue scale (VAS) was used where 0 represented no pain at all and 10 represented the greatest pain. VAS scale was measured before and after 24 h of each treatment (Table 8). PBM therapy was the treatment of choice. For the management of taste alteration, one session of PBM therapy was carried out each 24 h for five consecutive days, and the same procedure was repeated after 48 h. The irradiated areas were 10 points on the dorsum of the tongue, three points on the right lateral of the tongue, and three points on the left lateral of the tongue (Figure 3). Diode laser 635 nm (smart M Pro, Lasotronix, Poland) was used with an energy density of 3 J/cm^2^, 30 s of irradiation, output power of 100 mW, continuous and contact mode (Table 9). For the management of burning mouth sensation, diode laser 635 nm was used with the same previous parameters on the following areas: Three points on the tongue, four points on the lateral border of the tongue, 10 points on the dorsal surface of the tongue, eight points on the buccal mucosa, five points on the labial mucosa, eight points on the hard palate, three points on the soft palate, three points by sextant on the gingiva (Table 9). After PBM therapy, the taste alteration score was 5/5 (Table 8). According to the results, PBM can be considered as an effective approach for the management of taste alteration in cancer patient.

## 3. Discussion

In recent years, much knowledge has been gained on the PBM therapy mechanism of action after a plethora of laboratory, animal, and human studies [23,24,25]. In fact, over 100 phase III randomized controlled trials and over 1000 laboratory studies have studied the effects of photobiomodulation in different branches of medicine [24,25]. The biological modulation due to light therapy is the conversion of luminous energy to metabolic energy, which will lead to the modulation of cell functioning, and it happens when the near-infrared and infrared light reaches the targeted tissue. Photoacceptors, also called chromophores, are molecules found in nearly all living cells that absorb light energy and cause a change in cell function [26]. Chromophores typically absorb very specific wavelengths of light and reflect others, and it is the absorption of energy by chromophores during light irradiation that determines the specific biological responses [26]. Furthermore, it is now established that PBM acts principally on the chromophore cytochrome c oxidase (CcO) and the intracellular water. The CcO that is found in the mitochondria is the terminal enzyme of the electron transport chain, intermediating the electron transfer from cytochrome c to molecular oxygen. Therefore, CcO is implicated in the ATP production, which means that a stimulation of the CcO will lead to a stimulation in the ATP production. It was found that CcO acts as a photo-acceptor and transducer of photo-signals in the red and near-infrared regions of the light spectrum [27]. An increase in intracellular ATP is one of the most frequent and significant findings after PBM both in vitro and in vivo. Therefore, the stimulated synthesis of ATP is caused by an increased activity of CcO when activated by PBM. In addition, photobiomodulation induces a redox effect by stimulating a short and transient activation of the reactive oxygen species (ROS). Large doses of light, and even more particularly blue light, leads to the production of ROS, and it is well known that mitochondria are one of the most important sources of ROS; therefore, the PBM is somehow implicated in the induction of redox effects. Moreover, PBM is implicated in the activation of transcription factors and signaling pathways, since many of the secondary mediators of PBM, like the reactive oxygen species, are able to activate transcription factors and signaling pathways [11].

Acute and chronic oral complication as a side effect of cancer therapy represents a serious clinical challenge and affects largely the quality of the life of the cancer patients. The fact that photobiomodulation has shown to be efficient in the curative and preventive management of oral mucositis has led to a motivation for further studies to apply photobiomodulation therapy in the other, less frequent, oral complications of cancer therapy [28]. Furthermore, studies are being conducted on the efficacy of PBM in the reduction of neuropathy symptoms and on the possible neuro-regenerative effects. A prospective, randomized, placebo-controlled study with seven breast cancer patients with a chemotherapy-induced peripheral neuropathy assessed the efficacy of PBM. Based on the study, there seems to be a tendency towards the prevention of chemotherapy-induced peripheral neuropathy with the photobiomodulation therapy [29]. In addition, a randomized, sham-controlled clinical trial on 70 patients showed that the chemotherapy-induced peripheral neuropathy was significantly reduced, there was no significant reduction in the sham group, and that the addition of physiotherapy had no positive income [30].

In this case series, the oral complications managed were oral mucositis, dysphagia, oral dryness, taste alteration, and burning mouth sensation. To the extent of our knowledge, there are only few published studies on PBM for the management of dysphagia, oral dryness, burning mouth sensation, and taste alteration in cancer patients. A review article published by an international multidisciplinary panel of clinicians and researchers with expertise in the area of supportive care in cancer and PBM clinical application and dosimetry proposed a new treatment protocol to be used specifically for each of the oral complications [19,31]. Therefore, in order to optimize the parameters in this case series, and with the aim of having a better outcome, the suggested parameters and the treatment protocol by the international multidisciplinary panel were followed. In this case series, a significant improvement of taste perception and a significant decrease in the burning mouth sensation was noticed after 10 sessions of the treatment. In addition, a significant reduction of swallowing impairment after five sessions was noted, and an increase in the whole resting and stimulated saliva quantity was noted after five sessions. These results indicate that PBM therapy within the suggested parameters and treatment protocol can be considered as a promising approach for the management of the oral complications due to cancer therapy.

Despite the frequency of these oral complications in cancer patients, the pathophysiology of these complications is not fully understood. Dysphagia can be due to anatomical, mechanical, or neurological changes affecting any structure from the lips to the gastric cardia [32]. Dysgeusia during cancer therapy is usually attributed to the destruction of the dividing taste bud cells and olfactory receptor cells that are mostly found on the tongue, which explains the recommendations to use the PBM therapy on the tongue [33]. The oral dryness and hyposalivation are usually associated with the irradiation of the salivary glands and the loss of their function [34]. In some cases, apoptosis in parotid glands can be seen if the doses are relatively high [35]. This process is *p* 53-dependent [34].

Oral mucositis (OM) is the most frequent complication of cancer therapy, having a frequency of appearance in 80% of patients under high-dose chemotherapy and 80% of patients undergoing head and neck radiotherapy. A large number of studies have suggested the effectiveness of PBM in the management of OM [19]. In this matter, the levels of evidence for the recommendations by the MASCC/ISOO on the use of PBM in patients receiving hematopoietic stem cell transplantation (HSCT) in addition to head and neck cancer (HNC) patients are respectively set at II and III [36]. In fact, the panel of experts recommended the application of PBM as a preventive measure of OM in patients undergoing high-dose CT with or without total body irradiation before HSCT using the following parameters: Wavelength at 650 nm, power of 40 mW, and each cm^2^ treated with the required time to a tissue energy dose of 2 J/cm^2^ [19]. As for the HNC patients, with the lower level of evidence, the panel “suggests” the use of PBM (wavelength = 632 nm) as an OM prevention in patients undergoing radiation therapy (RT) without concurrent CT [36]. Concerning the burning mouth sensation, a recent meta-analysis that included 10 studies concluded that PBM therapy seems to be effective in the management of burning mouth sensation [37]. However, it is worth noting that the study did not include any cancer patients. To the best of our knowledge, this case series is the only study available in literature discussing the use of photobiomodulation for the management of the burning mouth sensation in a cancer patient. Furthermore, the problem of taste alteration as a consequence of head and neck radiotherapy and/or high dose chemotherapy has been recently highlighted as it was suggested to uniform the terminology of such complication to dysgeusia and taste alteration instead of using the following terms: Ageusia and taste dysfunction [38]. For this reason, in this case series, only the terms “dysgeusia” and “taste alteration” were used [38].

Another important issue is the standardization of the PBM treatment protocol and the laser irradiation parameters [39]. The use of infrared or near-infrared laser light is not only what it takes to have positive results for the management of oral complications. Several factors, parameters, and conditions influence the therapeutic effects of PBM, including fluence, irradiance, treatment timing and repetition pulsing, and wavelength. The wavelength, power density, energy density, and time of exposure must be properly adjusted in order to have a successful treatment [31,39]. Again, this is why in this case series the parameters that we used were those suggested in previous published review articles by experts in the field of PBM and supportive care [19,31].

Lastly, it is important to indicate that the North American Association for photobiomodulation therapy (NAALT) do not recommend the PBM therapy over an active tumor site to avoid any possible effect PBM therapy might have on active cancer sites, notably from the belief that there is a risk of transformation of premalignant cells or stimulation of active cancer cells [40]. Moreover, a systematic review suggests, based on 27 articles that meet the criteria, that the use of PBM in the prevention and management of cancer treatment toxicities does not lead to the development of tumor safety issues [41]. In accordance with the previous findings, a retrospective study of the safety of PBM in patients with head and neck cancer showed no effect of PBM upon overall survival, time to local recurrences, and disease-free survival of patients with head and neck cancer treated with radiotherapy with/without chemotherapy [42].

This case series suggest the effectiveness of PBM therapy in the management of oral mucositis, dysphagia, oral dryness, taste alteration, and burning mouth sensation due to cancer therapy. However, the absence of a control group and the relatively small number of included patients can be considered as a limitation of the findings. Hence, randomized clinical trials with a control group and a larger number of included patients using the same treatment protocol and parameters is recommended.

## 4. Conclusions

Within the limitations of the study, photobiomodulation therapy with the specific parameters and treatment protocol used in this study can be considered effective in the management of oral mucositis, dysphagia, oral dryness, taste alteration, and burning mouth sensation due to cancer therapy. Further studies need to be done to confirm its effectiveness and to identify the optimal parameters and treatment protocol.

## Figures and Tables

**Table 1:** National Cancer Institute assessment scale for oral mucositis.

Grade	Description
Grade 0 (none)	None
Grade 1 (mild)	Painless ulcers, erythema, or mild soreness in the absence of lesions
Grade 2 (moderate)	Painful erythema, edema, or ulcers but eating or swallowing possible
Grade 3 (severe)	Painful erythema, edema, or ulcers requiring IV hydration.
Grade 4 (life-threatening)	Severe ulceration or requiring parenteral or enteral nutritional support or prophylactic intubation.
Grade 5 (death)	Death related to toxicity.

**Table 2:** Functional outcome swallowing scale for staging oropharyngeal dysphagia proposed by John R. Salassa in the 39th annual meeting of the American Society for Head and Neck Surgery.

Stage	Stage Criteria
Stage 0	Normal physiological function and asymptomatic.
Stage I	Normal physiological function but with episodic or daily symptoms of dysphagia such as reflux symptoms, globus, odynophagia, repetitive swallow, throat-clearing habit, difficulty chewing, minor oral incompetence, sensation of food getting stuck in the throat or esophagus.
Stage II	Compensated abnormal function manifested by significant dietary modifications or prolonged mealtime. Weight is stable, cough is absent or occasional, aspiration is absent or occasional and mild.
Stage III	Decompensated abnormal function manifested by weight loss of 10% or loss of body weight over 6 months due to dysphagia, or frequent cough, gagging, or aspiration during meals. Aspiration may be mild or moderate. Patients in this stage are unstable in terms of nutrition or respiratory status. Pulmonary complications have not occurred, but the patient is at risk.
Stage IV	Severely decompensated abnormal function manifested by weight loss of more than 10% of body weight over 6 months due to dysphagia, or severe aspiration. Non-oral feeding recommended for most (>50%) of nutrition. Patients in this stage are nearly complete failures at swallowing and may safely swallow only under strictly defined conditions, which do not meet their nutritional needs.
Stage V	Nonoral feeding for all nutrition. Patients in this stage are complete failures at swallowing. They are different from stage IV in that they cannot swallow anything safely.

**Table 3:** Photobiomodulation therapy for the management of oral mucositis parameters: Applications and treatment protocol.

Irradiation	Treatment Area	Parameters
Intraoral	Four points on the tongue and two on the oropharynx.	Diode laser 635 nm, energy density of 3 J/cm^2^, time of 30 s per spot, output power of 100 mW in a continuous and contact mode.
Extraoral	Lips, cutaneous surface corresponding to the buccal mucosae, bilateral cervical lymphatic chain.	

**Table 4:** Photobiomodulation therapy for the management of dysphagia parameters: Applications and treatment protocol.

Irradiation	Treatment Area	Parameters
Intraoral	Four points on the soft palate, four points on the oropharynx. Bilaterally, four points to soft palate and onto oropharynx.	Wavelength of 635 nm, 3 J/cm^2^ for 10 s on each point, 100 mW, continuous and contact mode.
Extraoral	Lateral and ventral pharynx and larynx. Midline neck and lateral neck anterior to sternocleidomastoid muscle.	

**Table 5:** Results of the assessments of oral mucositis using the national cancer and dysphagia using the functional outcome swallowing scale for staging oropharyngeal dysphagia.

Assessment Method	T*i*	T1	T2	T3	T4	T5
FOSS scale	2	2	1	1	0	0
NCI scale	2	2	1	0	0	0

T*i* = before treatment, T1 = after 24 h of the first session, T2 = after 24 h of the second session, T3 = 24 h after the third session, T4 = 24 h after the fourth session, T5 = 24 h after the fifth session, FOSS = functional outcome swallowing scale for staging oropharyngeal dysphagia, NCI= national cancer institute. Oral mucositis measurements were made according to the National Cancer Institute.

**Table 6:** Photobiomodulation therapy for the management of oral dryness: Parameters and treatment protocol.

Oral Complication	Treated Area	Parameters
Oral dryness	Intraoral application: 10 points on the major salivary glands: Parotid and submandibular glands. Minor salivary glands in each side. 10 points on the dorsal aspect of the tongue.	Diode laser 635 nm. Energy density of 3 J/cm^2^ for 30 s, output power of 100 mW, continuous and contact mode.

**Table 7:** Assessment of the quantity of completely resting and stimulated saliva before, during, and after treatment (Q-sal, mL/min).

Quantity of Saliva	T*i*	T1	T2	T3	T4	T5
Before stimulation	0.03	0.03	0.05	0.07	0.12	0.2
After stimulation	0.1	0.15	0.15	0.3	0.3	0.4

T*i* = before treatment, T1 = after 24 h of the first session, T2 = after 24 h of the second session, T3 = 24 h after the third session, T4 = 24 h after the fourth session, T5 = 24 h after the fifth session.

**Table 8:** Assessment of the quantity of whole resting and stimulated saliva before, during and after treatment (Q-sal, mL/min).

Assessment Method	T*i*	T1	T2	T3	T4	T5	No Treatment for 48 h	T6	T7	T8	T9	T10
ISO 3972: 2011 score for taste alteration	0	0	1	1	2	2	2	2	4	4	5	5
Visual analogue scale for burning mouth sensation		7	7	6	6	4	4	4	4	2	1	0

T*i* = before treatment, T1 = after 24 h of the first session, T2 = after 24 h of the second session, T3 = 24 h after the third session, T4 = 24 h after the fourth session, T5 = 24 h after the fifth session…, T10 = 24 h after the 10th session.

**Table 9:** Photobiomodulation therapy for the management of taste alteration.

Oral Complicatin	Zone Irradiated	Parameters
Taste alteration	10 points on the dorsum of the tongue Three points on the right lateral Three points on the left lateral aspect of the tongue	Diode laser 635 nm. Energy density of 3 J/cm^2^ for 30 s, output power of 100 mW, continuous mode contact mode
Burning mouth sensation	Tip of the tongue: Three points Lateral border of the tongue: Four points Dorsal surface of the tongue: 10 points Buccal mucosa: Eight points Labial mucosa: Five points Hard palate: Eight points Soft palate: Three points Gingiva: Three points by sextant	
